# Raman Spectroscopy for Testing Wood Pellets

**DOI:** 10.3390/mps9010003

**Published:** 2025-12-21

**Authors:** Tereza Zemánková, Martin Kizovský, Zdeněk Pilát, Pavlína Modlitbová, Jan Ježek, Martin Šiler, Ota Samek

**Affiliations:** 1 Institute of Scientific Instruments of the Czech Academy of Sciences, v. v. i., Královopolská 147, 612 00 Brno, Czech Republic; martink@isibrno.cz (M.K.); pilat@isibrno.cz (Z.P.); modlitba@isibrno.cz (P.M.); jezek@isibrno.cz (J.J.); siler@isibrno.cz (M.Š.); 2 Department of Optics, Faculty of Science, Palacký University, 17. listopadu 12, 771 46 Olomouc, Czech Republic

**Keywords:** Raman spectroscopy, wood pellets, biomass, lignin, cellulose, forestry residues, chemical composition, rapid analysis, portable Raman, quality control

## Abstract

The creation of bioenergy based on the biomass wood pellet industry, which accounts for the majority of the global biomass supply, is one of the most common and important ways to utilize waste wood, wood dust, and other byproducts of wood manufacturing, known as forestry residues. Pellet production processes might greatly benefit from fast monitoring systems that may allow for at least a semi-quantitative measurement of crucial parameters such as lignin and cellulose. The determination of lignin and cellulose is complicated and time-consuming because it usually requires time-demanding and labor-intensive sample preparation. This, however, might be a crucial problem. In this context, the application of Raman spectroscopic techniques is considered a promising approach, as it enables rapid, reliable, and label-free analysis of wood pellets, providing information about the chemical composition of the biomass, specifically lignin and cellulose. The purpose of this article is to report on the application of Raman spectroscopy exemplified by the detection of the lignin/cellulose ratio. In our methodological approach, we integrated the area under the selected Raman bands to avoid a large scatter of data when only the intensities of the bands were used. Moreover, the acquired Raman spectra displayed very strong signals from both substances, which contributes to the feasibility of the analysis even with a portable instrument. This study is expected to be of assistance in situations when the monitoring of the chemical changes and the quick inspection of pellets are required in near real time, online, and in situ.

## 1. Introduction

It has been proven that the technique of Raman spectroscopy may be regarded as the method of choice for many studies of biomass and wood, while the Raman scattering of water is weak, thus allowing this technique to be applied to both fresh and dried plant material [1,2,3,4]. Raman microspectroscopy enables the analysis of biological tissues from the leaf level [5,6] down to the cellular or subcellular level [7,8]. It has been established as a non-invasive analytical tool for chemical and structural analyses of wood [9], including the imaging analysis of the cell-wall structure [1,2,3].

Raman spectroscopy may be employed for a wide range of biotechnological studies of microorganisms, cells, and biological samples in general [10,11,12,13,14,15,16,17]. Two recent reviews provide valuable information on Raman spectroscopy studies of the molecular complexes in biological specimens [18,19]. In addition, a reasonably detailed database of Raman features encountered in biological samples was published [14].

In one study, Raman microscopy was used to quantify the lignin contents of various wood tissues in Japanese wood species. This study revealed that the lignin contents in wood varied depending on species or tissues [20]. The utilization of Raman spectroscopy in wood biomass research was presented by Anni Lähdetie [21] in her doctoral thesis. This detailed study illustrates the research in the analysis of the structure and reactions of lignin.

Moreover, it has been shown in several studies that techniques based on infrared spectroscopy (IR) are capable of identification and discrimination of raw materials used in pellet production [22] and also for the identification of five Brazilian native wood species [23]. In the study of Agarwal et al. [24], cellulose crystallinities of a large number of lignocelluloses were estimated using the near-IR Fourier transform (FT)-Raman method. The authors pointed out the role of lignin and cellulose. The prediction of hardwood and softwood contents in blend samples by means of FTIR was targeted in a study by Duca et al. [25]. In the report by Gao et al., Raman spectra were excited using a 1064 nm laser, thus avoiding the impact of water and autofluorescence on near-IR signals for evaluation of lignin from biomass [26].

For reviews more focused on advances in chemical imaging of lignocellulosic biomass, the readers are directed to the three excellent reviews [27,28,29] focusing on IR, fluorescence, and Raman microspectroscopy, which include tip-enhanced Raman spectroscopy, spontaneous Raman scattering, stimulated Raman spectroscopy, and coherent anti-Stokes Raman spectroscopy. For the application of Raman spectroscopy in wood characterization and structural evolution monitoring during the functionalization and imaging of lignocellulosic materials, readers may be directed to the recent review [30]. Recently, Sharma et al. proposed a novel approach that leverages attenuated total reflectance FTIR combined with machine learning to enhance the accuracy and efficiency of wood species identification [31]. The group focused on species that might be potentially subject to illegal felling and trade.

In modern Raman spectroscopy, the benefits of this method regarding the wood samples are at the forefront of research into combating problems such as the identification of the chemical composition of pellets. Raman spectroscopy demonstrates clear benefits over the existing techniques that target chemical analysis and include, e.g., laser ablation atomic emission spectroscopy, X-ray fluorescence, and mass spectrometry-based techniques. Raman spectroscopy excels in the speed of the identification process, which is particularly important when considering online analysis.

Raman spectra of wood tissues particularly reflect vibrational features of lignin, cellulose, and hemicelluloses [3]. The contribution of hemicelluloses to the Raman spectra overlaps with that of cellulose. Due to their more amorphous nature, Raman bands of hemicelluloses are broader and significantly weaker in intensity [3].

Up to one-third of plant biomass consists of lignin, which is the second most abundant polymer on Earth. Another basic structural component of plants is cellulose. Consequently, the main aim of this study is to monitor and identify the changes in lignin/cellulose (Li/Ce) ratio in relation to the different producers of wood pellets.

Lignin is synthesized in xylem tissue and plays an important role in cell wall formation. It provides a hydrophobic surface, allowing vascular plants to transport water effectively to the heights of several meters. The incorporation of lignin helps in binding the cellulose microfibrils. Subsequently, it improves the cell-wall structural rigidity and durability [32,33].

The objectives of this study were to evaluate the potential for using Raman spectroscopy in analyzing the changes in the Li/Ce ratio in wood pellets, thereby reflecting the degree of lignification, which directly translates to the pellets’ heating value. Raman spectroscopic records of the Li/Ce ratios were therefore obtained from individual pellets provided by different producers, and the datasets were evaluated via principal component analysis (PCA). These findings also contribute to the characterization of the chemical properties of input wood as a function of its origin [34,35,36,37,38].

The Li/Ce ratio is crucial for a biomass’s heating value, which, in turn, translates to the commercial production of wood pellets. In general, the heating value of the lignocellulosic fuels rises with an increase in their lignin content. Lignin has a higher calorific value (23–27 MJ/kg) than cellulose (19 MJ/kg) [39]. While the high lignin content is desirable for its heating value, it also acts as a natural binder, which may improve the durability of the pellets [40]. Hence, an increase in the lignin content of wood is associated with an improvement of pellet quality, mostly regarding the heating value, bulk density, durability, and the ash content [41]. Consequently, when controlling the pellet production quality—namely the lignin content—it is a crucial parameter in pellet production.

In the present study, we exploit Raman spectroscopy for the rapid estimation of Li/Ce ratio, directly applied to the study of wood pellets. Thus, the use of Raman spectroscopy is a promising solution for gaining new and additional information in pellet manufacturing.

## 2. Materials and Methods

The investigation presented in this specific report expands on the findings from our earlier publication on Raman spectroscopy of wood samples directly measured on selected samples, in which we explored the ability of Raman spectroscopy combined with laser-induced breakdown spectroscopy to detect the Li/Ce ratios [42].

**Figure 1:**
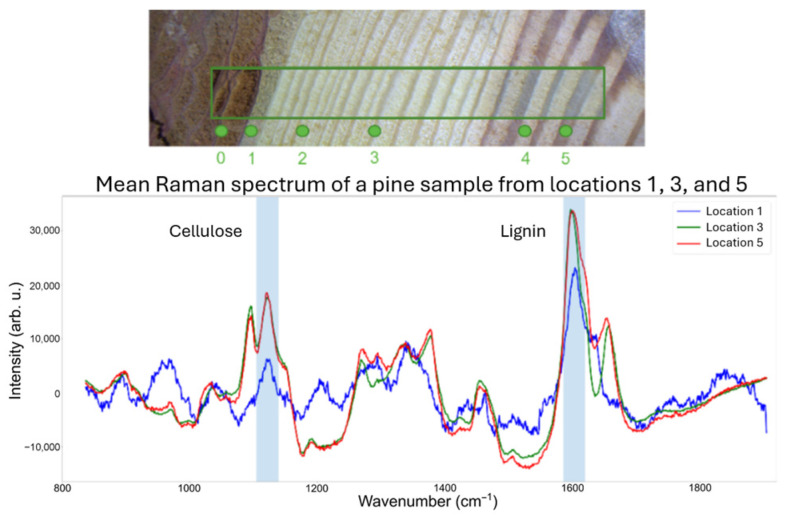
Illustration of the distribution for lignin and cellulose in a real wood sample (pine) obtained by Raman spectroscopy.

In this study, pieces of wood cut from a pine trunk were utilized as samples, where the annual rings are visible in Figure 1. For demonstration and illustration purposes, we present Raman spectra from the three different locations that clearly show the inhomogeneous distribution of lignin and cellulose within the wood cut of a pine trunk. It shows that the distribution of lignin and cellulose strongly depends on the position, e.g., bark and diverse annual rings. Consequently, these inhomogeneities are transferred into the wood pellets, as pine is often used as the raw material and all parts of the input wood are ground and mixed to form the wood pellets.

However, in contrast to analyzing raw wood samples, the study presented here focuses specifically on the investigation of the wood pellets themselves. Biomass pellet fuel is one of the most common and important ways of utilizing biomass energy. The use of diverse biomass resources with different properties for pelletizing is a current research hotspot. The wood pellet production process involves the preparation of the raw material, which is then dried and ground into sawdust. Afterwards, the sawdust is compressed into a pellet mill, where the heat from friction plasticizes the natural lignin in the wood, acting as a binder. Finally, the hot pellets are cooled and packaged for storage or distribution [43].

In the present study, we exploited the spot-spectra recording for rapid mapping of the parts of the pellets. Specifically, we investigated the surface of the pellets to remain within the focal plane of the exciting laser and imaging optics. In this way, the heterogeneity of pellets, introduced in the process of manufacturing, might be measured, visualized, and analyzed. We used the large sets of data generated from the pellets from eight different producers (50 recorded spectra from pellets of one producer were included; 400 spectra were acquired in total). Consequently, differences between different pellet manufacturers were identified by using PCA.

In our study, we tested wood pellets from eight different producers. A list of all manufacturers may be provided on request. We would like to note that the chemical composition of biomass has a significant influence on the fuel quality, including the heating value [44,45,46]. According to the manufacturers’ information, all pellets should be manufactured from pure dried spruce/pine sawdust without bark and chemical binders with the certification of ENplus A1. The pellets are suitable for all types of stoves, boilers, and fireplaces designed for firewood pellets, as well as for the automatic coal-fired boilers.

When characterizing different samples (wood, bacterial colonies) via using the spectroscopic techniques, specifically Raman spectroscopy, one is normally faced with the problem of spatial inhomogeneity of the sample. On the one hand, this allows a researcher to evaluate different parts of a sample (such as annual rings, etc.) depending on environmental conditions, such as a function of the position. On the other hand, it might hinder the clear identification of a particular sample because of its roughness and related problems with refocusing using the mapping regime. Nevertheless, in wood pellet samples, the heterogeneity is low, and the surface of the pellets is very smooth. This enables the determination of the Li/Ce ratio from the multipoint measurements.

Both the particular species and some relevant molecular complexes might be identified this way. This type of spot investigation of species and/or compound identification may collect sufficient or complete (multispectral) information about the entire sample, including the likely inhomogeneous distribution across the sample’s dimensions.

Thus, building up a data set may be achieved over reasonably short time intervals; e.g., a single pellet may be analyzed very quickly (in just a few minutes). With the rapidly acquired spectra–up to several tens of spectra–the analysis and identification have become possible as well, using the standard chemometric techniques. At this stage, the required amount of data is sufficient for the complexity of the problem.

### 2.1. Raman Spectroscopy Measurement

We used the inVia^™^ Renishaw Raman spectrometer (Renishaw, Wotton-under-Edge, UK) for all the Raman spectroscopic measurements. To acquire the Raman spectra, we implemented the laser with an excitation wavelength of 785 nm and the microscope objective Leica N Plan EPI (Leica, Wetzlar, Germany) with a magnification of 50 and a numerical aperture of 0.75. The working distance is approximately 0.5 mm. Our inVia^™^ Renishaw system is equipped with a line laser, which provides a laser spot area of approximately 2 μm × 10 μm. We used a grating of 1200 gr/mm and the RenCam CCD detector. Laser power for the Raman excitation was about 10 mW on the sample. The power was decreased in order to prevent the oversaturation of the detector. The values of laser power were obtained by the Thorlabs PM100D power meter (Thorlabs, Newton, NJ, USA); the measurements were acquired at the focal plane of the laser. Each spectrum is in the range of 800–1800 cm^−1^, which is determined by the grating of the instrument and covers all the expected peaks of the analytes. The acquisition of every spectrum took 10 s in total; the integration time was 1 s with 10 accumulations per spectrum. Before each acquisition, the excitation laser was focused on the pellet surface. All spectra have been processed in the same way. The Savitzky-Golay algorithm was utilized to smooth the spectra (order: 2, frame length: 7). The background was removed with the rolling-circle filtering (radius: 800 cm^−1^, passes: 10). The spectra processing was performed with an in-house created software based on MATLAB 2018b (MathWorks, Natick, MA, USA).

### 2.2. Handheld/Portable Raman Spectrometer

Handheld Raman spectroscopy is usable for analyzing wood samples/pellets, and may be used for quality control. Handheld instrumentation may provide rapid, non-destructive analysis, especially when combined with chemometric methods to interpret the data. Measurements with a homemade portable Raman spectrometer, built for the fieldwork at our institute, were performed in our laboratory. The instrument is equipped with a laser (butterfly diode coupled into a single-mode fiber) of a 785 nm wavelength and a maximum power of 200 mW. This is fed into the measuring head, from which the measured signal is transmitted via the multimode fiber to a spectrometer with a Czerny-Turner grating configuration designed by our institution, with the grating of 1200 gr/mm. The detection camera is equipped with a Hamamatsu chip (model S16011), with 1064 × 64 pixels and increased efficiency in the near-IR spectrum.

### 2.3. Method of Choice

In the first step of the rapid pellet testing, we explored a method that determines the ratio between lignin and cellulose single bands [47,48]. The Li/Ce ratio provides information about the wood used for pellet production and ensures the use of the correct raw materials—pure dried spruce/pine sawdust without bark and chemical binders. Also, the calorific value of the pellets depends on this ratio. If the measurement is applied online during the manufacturing of pellets, the effects of different processing methods, the determination of the quality of the pellets, and the monitoring of the chemical changes during the processes are translated into this ratio.

Agarwal et al. [47] plotted the ratio of lignin to cellulose concentration by using the three plots generated for the three bands of cellulose (380, 1098, and 2900 cm^−1^). All three bands exhibited a similar pattern in regard to the variation in the value of the ratio. Vitek et al. [48] used a ratio of lignin to 1098 cm^−1^ peak of cellulose. Hence, these previous findings of different groups lead to further support for this methodology being used in our study.

As already mentioned, Raman spectroscopy provides molecular-specific scattered light that offers valuable information on the compositional features of wood tissues. The distinct spectral features of lignin and cellulose enable the separate analysis of their contributions.

### 2.4. Lignin Contribution

Lignin’s spectral contributions are primarily associated with the strongest Raman band near 1600 cm^−1^, which is assigned to aryl ring breathing (symmetric). This signal intensity is proportional to the concentration of phenyl-containing molecules, including lignin. In the spectrum of pine, the band at approximately 1600 cm^−1^ is the most intense Raman band derived from lignin.

### 2.5. Cellulose/Carbohydrate Contribution

Cellulose is a highly ordered and crystalline polymer, resulting in sharp and intense bands in the Raman spectrum. The key cellulose bands are often discovered in the range of 1095 cm^−1^ (a strong heavy atom C-C and C-O stretching) and 1123 cm^−1^ (a strong heavy atom C-C and C-O stretching).

Since the spectral regions where lignin contributes (e.g., around 1600 cm^−1^) do not overlap significantly with the characteristic regions of cellulose contribution (e.g., around 1095 cm^−1^), the Raman spectrum of wood may be primarily interpreted in terms of cellulose and lignin. Consequently, the Li/Ce ratio is calculated as the ratio of the peak heights in these positions (1600 cm^−1^/1096 cm^−1^).

The Li/Ce ratio is a crucial parameter for characterizing the plant biomass and lignocellulosic materials. Studies using Raman spectroscopy have revealed significant differences in the Li/Ce ratio based on a tree type, tissue composition, and environmental factors [48].

### 2.6. Methodology

**Figure 2:**
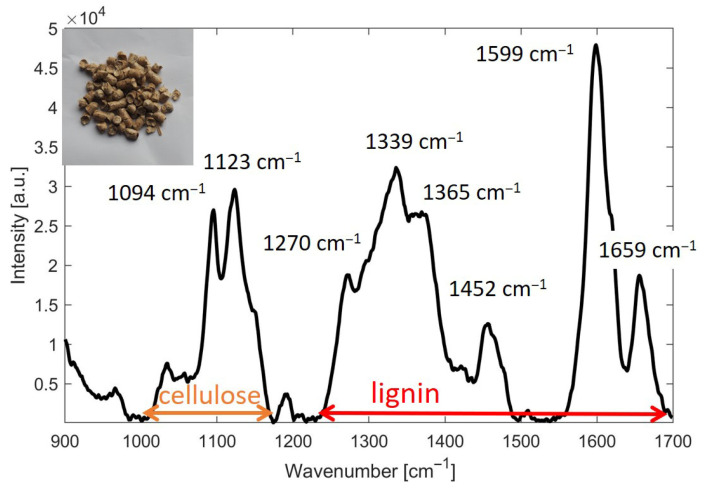
Illustration of the ranges of lignin and cellulose used for estimating the Li/Ce ratio based on Raman spectroscopic data from wood pellets. Assignment of Raman peaks for lignin and cellulose is according to Gierlinger et al. [3].

Since the evaluation of the Li/Ce ratio in wood pellets, based on the intensity of Raman transitions described in the previous sections, was subject to a large error due to the inhomogeneity of the pellets, we in turn decided to choose a different methodology. This methodology is based on the integration of the area under the selected Raman bands belonging to lignin and cellulose. The following spectral ranges were selected: (i) lignin: 1250–1700 cm^−1^ and (ii) cellulose: 980–1150 cm^−1^. A graphical representation of these two ranges is shown in Figure 2.

### 2.7. Data Analysis

In our study, we chose PCA, a statistical procedure relying on an orthogonal transformation to convert the measured Raman spectra into a set of linearly uncorrelated variables, the so-called principal components. We used the uncentered PCA implementation provided by MATLAB. Each spectrum is represented as a single point; PCA is often utilized to decrease the number of variables needed to describe a dataset. In our study, the first two scores (PCA components) describe almost 99% of the total variance present in measured spectra. To visualize and determine the boundaries between the datasets coming from different species, the so-called Mahalanobis distance was employed for better visualization (drawn as ellipses). The Mahalanobis distance describes the distance of a point to the center of a random distribution [49]. Another technique employed in conjunction with PCA was the linear discriminant analysis (LDA). This is a supervised method that aims to find the projection that best separates the classes in the data; this might, in turn, better visualize our findings [50].

## 3. Results and Discussion

**Figure 3:**
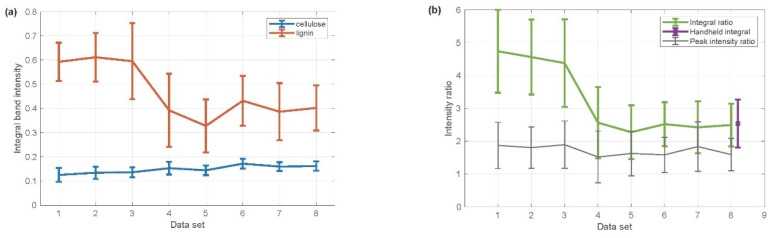
(**a**) The integrated area below the two selected ranges for cellulose (blue trace) and lignin (red trace) for eight different pellet manufacturers. (**b**) The Li/Ce ratio calculated from the integrated areas (top green trace) and the Li/Ce ratio computed from the ratio of a given intensity (bottom black trace). Consequently, this highlights the improvement of the ratio calculated from the integrated areas over the ratio of a peak intensity—the difference between the first three samples and the rest is more pronounced. On the right side of the figure, an additional error bar (purple) is applied for the handheld measurements to compare the handheld instrument with the lab-based instrument (see the inset in the top right corner).

Wood pellets from eight different producers were selected for the Raman-based Li/Ce ratio. From each producer, data were obtained from five different wood pellets (ten spectra from each pellet). Thus, 50 Raman spectra acquired from one manufacturer might be further studied in detail. In total, 400 Raman spectra were recorded with a lab-based Raman instrument (Figure 3).

Using our in-house software program, the two integration areas (lignin and cellulose), described in Section 2.6, were calculated and plotted for each manufacturer. Figure 3a shows the data calculated separately for lignin and cellulose, while Figure 3b demonstrates the Li/Ce ratio computed from the integrated areas based on Figure 3a. From Figure 3 it may be deduced that the pellets from the first trio of producers contain more lignin than pellets produced by the following manufacturers. To highlight the advantage of an integrated area used for the ratio calculation, the Li/Ce ratio, determined from the ratio of the given intensity of lignin and cellulose Raman peaks, was also included.

**Figure 4:**
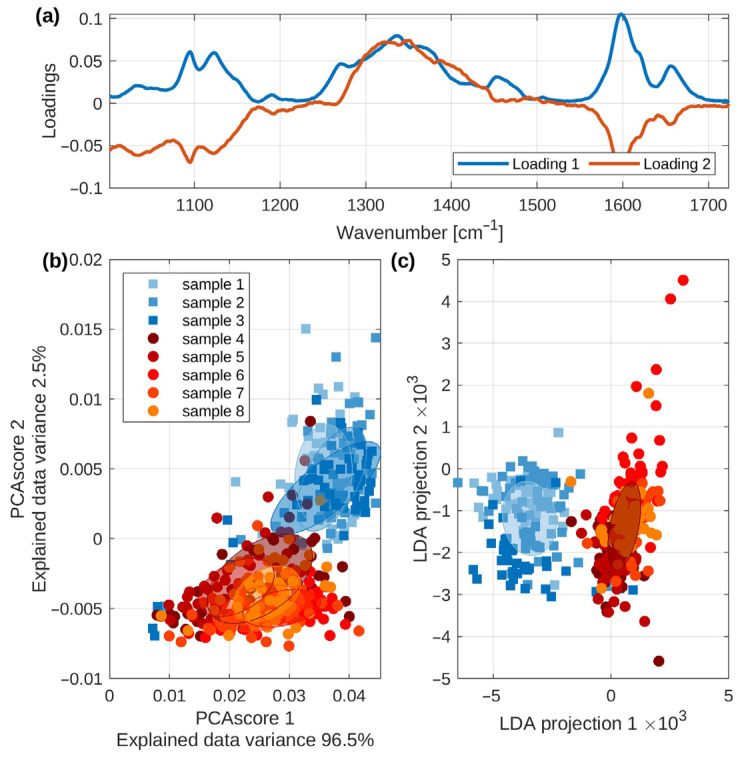
(**a**) The PCA loadings manifest which bands contribute to the changes presented at the PCA plot. For the PCA calculations, the spectra ranging from 100 cm^−1^ to 1700 cm^−1^ were used. (**b**) The PCA plot, based on all the data obtained from our experiments, shows eight different pellet manufacturers. (**c**) The LDA plot. Moreover, Figure 4 shows the evidence that PC-loading presentations constitute a valuable tool for estimating the relative contributions from different molecules present in the sample. In this particular case, mainly the contributions from lignin and cellulose are depicted.

Figure 4a shows the first trio of loadings of the PCA transform. It may be seen that these curves differ, especially in the spectral regions of cellulose as well as lignin presented in Figure 2. The PC loadings in Raman spectra manifest which Raman peaks (variables) are the most important for the sample differentiation along a principal component, essentially mapping the spectral contribution to the variance identified in the data. A plot of the loadings against the wavenumber results in a spectrum-like graph revealing the underlying chemical differences, such as changes in lignin and cellulose, that are driving the separation between the different sample groups.

The PCA score plot is depicted in Figure 4b. Each set of spectra corresponding to a single sample is rendered in a different color and symbol. Following the previous discussion (Figure 3), we marked the high-lignin-response samples (samples 1–3) with squares of different shades of blue color and the lower-lignin-response samples with circles of shades of yellow/red colors.

In addition, we marked the data corresponding to each sample by a transparent elliptical region matching the Mahalanobis distance 1 (i.e., the unit circle in the coordinate system given by the eigenvectors of each sample covariance matrix) [49]. This allows the plain-sight perception of both types of samples to be nicely separated.

Nonetheless, the separation is not complete, while some outliers from both groups are located in the vicinity of the other samples’ “center of mass”. Therefore, we performed the LDA [50] on the first 10 PCA scores and visualized the projection of the first two LDA coordinates in Figure 4c. Please note that the LDA was performed assuming eight categories of data, i.e., the eight wooden pellet samples. Thus, the LDA, which, in principle, should maximize the distances between the data categories, distinctly separated the samples into two groups corresponding to the high and low lignin spectral response. There is still a small overlap between both sample types, as a few spectra projections of high-lignin samples overlap with the low-lignin data. However, these data points may be classified as outliers from the reasonably sized ensemble of spectral data (50 spectra per pellet sample, 400 spectra in total). The semi-transparent elliptical regions in Figure 4c reflect the Mahalanobis distance 1 for the merged samples: 1–3 (blue), 4–8 (red).

It is not unexpected that some scatter in the clustering for a given species is observed, suggesting the variance within the pellets. As mentioned before, the surface of each pellet shows a certain amount of heterogeneity. Spectral intensities slightly differ, considering the spectra associated with diverse points within the measurement area. However, the two principal components displayed here, PC1 and PC2, account for 99% of the total variance in our measurements.

Our presentations exploring the PCA plots promise a viable technique for Raman band/peak interpretation based on cellulose and lignin. However, further detailed work is necessary to unlock the full potential of this approach. Several studies are currently in progress to evaluate large data sets to further elucidate the variances visible in the PC-loadings with the aid of complementary chemometric tools.

**Figure 5:**
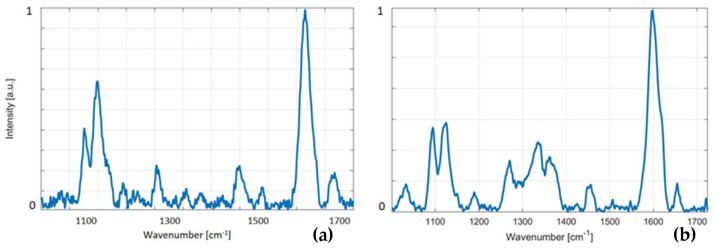
This figure demonstrates Raman spectra collected from a wood pellet by (**a**) a portable instrument and (**b**) a lab-based Raman instrument. We conclude that Raman spectra of good quality may be acquired in a few seconds, which, in turn, suggests that various handheld/portable systems may be used for this application.

Portable instrument: The use of our portable instrument for recording Raman spectra from wood pellets shows a small increase in the noise level and the resolution of spectra when compared to the lab-based instrument. The differences between the portable and lab-based Raman instruments are not significant for bands of our interest, due to a very high intensity in our dedicated analytes for wood pellets—lignin and cellulose. Figure 5 shows spectra from (a) a portable instrument and (b) a lab-based Raman instrument. This is just to demonstrate that spectra from a handheld instrument are of high quality and may be further utilized for the analysis. To show the data obtained from the handheld instrument, an analysis of spectra obtained by the handheld Raman instrument is presented in Figure 3b. We evaluated sample 8 so that the data from the handheld instrument might be directly (see Figure 3b) compared to the lab-based instrument. The data are shown as a “handheld integral” (see the inset in the top right corner) next to sample 8, which, in turn, demonstrates the feasibility of a handheld instrument in lignin diagnostics.

Thus, portable Raman devices may be routinely implemented for testing wood pellets in situations where the high sensitivity of a lab-based system is not necessary.

## 4. Conclusions

Results from a Raman spectroscopy study of eight pellet producers show the variability of the Li/Ce ratios, which, in turn, may affect the pellet parameters, such as heating value.

Our findings demonstrate that a quick and statistically significant analysis using Raman spectroscopy, resulting in an analysis performed in mere minutes, is feasible, offering detailed information on the pellets’ lignification. The lignification of wood pellets is highly dependent on the type of wood used, particularly its inherent lignin content, composition, and distribution. Different wood species have different lignin structures and percentages. All these aspects lead to the variations in pellet quality and strength. Thus, high-quality pellets should be manufactured from pure dried spruce/pine sawdust without bark and chemical binders; the least desirable variant results from barky, dirty, or treated woods, and agricultural waste (straw).

This suggests that Raman spectroscopy may be implemented in investigations of input wood entering the processing when the relationship between the lignin and cellulose is of interest. The introduced methodology may be applied by both the large- and small-scale wood pellet producers who often use a trial-and-error approach for determining the adequate blending of available wood processing residues and pelletizing parameters [51].

Moreover, in this report, we demonstrated that the handheld Raman systems might be utilized for on-site applications. This is due to the very high intensity of selected bands of lignin and cellulose, which offsets the disadvantages of a less sensitive instrument to be used in field conditions. Handheld instruments may be used to characterize the blends of residues when mixing the residues of different origins [51] directly online, in situ, or in near real time, which offers immediate feedback on the process of optimizing the wood pellet quality and, in turn, meeting the market standards.

## Data Availability

To access the Raman spectral data used in this publication, go to Raman Base, https://ramanbase.org [52] and follow the instructions.

## References

[1] Gierlinger N., Schwanninger M. (2007). The potential of Raman microscopy and Raman imaging in plant research. Spectroscopy.

[2] Gierlinger N., Keplinger T., Harrington M. (2012). Imaging of plant cell walls by confocal Raman microscopy. Nat. Protoc..

[3] Gierlinger N., Keplinger T., Harrington M., Schwanninger M., van de Ven T., Kadla J. (2013). Raman imaging of lignocellulosic feedstock. Cellulose Biomass Conversion 3.

[4] Gierlinger N. (2014). Revealing changes in molecular composition of plant cell walls on the micron-level by Raman mapping and vertex component analysis (VCA). Front. Plant Sci..

[5] Schulz H., Baranska M. (2007). Identification and quantification of valuable plant substances by IR and Raman spectroscopy. Vib. Spectrosc..

[6] Vítek P., Novotná K., Hodaňová P., Rapantová B., Klem K. (2017). Detection of herbicide effects on pigment composition and PSII photochemistry in Helianthus annuus by Raman spectroscopy and chlorophyll a fluorescence. Spectrochim. Acta A.

[7] Kaczor A., Pilarczyk M., Baranska M. (2014). Structural and spatial analysis of carotenoids in a single cell monitored by Raman spectroscopy. Optical Spectroscopy and Computational Methods in Biology and Medicine.

[8] Prats-Mateu B., Stefke B., Hauser M.-T., Gierlinger N. (2014). Elucidating structural and compositional changes in plant tissues and single cells by Raman spectroscopic imaging. Res. Gate.

[9] Agarwal U.P., Ralph S.A. (1997). FT-Raman spectroscopy of wood: Identifying contributions of lignin and carbohydrate polymers in the spectrum of black spruce (*Picea mariana*). Appl. Spectrosc..

[10] Schie I.W., Huser T. (2013). Methods and applications of Raman microspectroscopy tosingle-cell analysis. Appl. Spectrosc..

[11] Notingher I. (2007). Raman spectroscopy cell-based biosensors. Sensors.

[12] Prochazka D., Mazura M., Samek O., Rebrošová K., Pořízka P., Klus J., Prochazková P., Novotný J., Novotný K., Kaiser J. (2018). Combination of laser-induced breakdown spectroscopy and Raman spectroscopy for multivariate classification of bacteria. Spectrochim. Acta B.

[13] Almarashi J.F.M., Kapel N., Wilkinson T.S., Telle H.H. (2012). Raman spectroscopy of bacterial species and strains cultivated under reproducible conditions. J. Spectrosc..

[14] De Gelder J., De Gussem K., Vandenabeele P., Moens L. (2007). Reference database of Raman spectra of biological molecules. J. Raman Spectrosc..

[15] Brauchle E., Schenke-Layland K. (2012). Raman spectroscopy in biomedicine—Noninvasive in vitro analysis of cells and extracellular matrix components in tissues. Biotechnol. J..

[16] Li-Chan E. (1996). The applications of Raman spectroscopy in food science. Trends Food Sci. Technol..

[17] Samek O., Jonáš A., Pilát Z., Zemánek P., Nedbal L., Tříska J., Kotas P., Trtílek M. (2010). Raman microspectroscopy of individual algal cells: Sensing unsaturation of storage lipids in vivo. Sensors.

[18] Jones R.R., Hooper D.C., Zhang L., Wolverson D., Valev V.K. (2019). Raman techniques: Fundamentals and frontiers. Nanoscale Res. Lett..

[19] Orlando A., Franceschini F., Muscas C., Pidkova S., Bartoli M., Rovere M., Tagliaferro A. (2021). A Comprehensive review on Raman spectroscopy applications. Chemosensors.

[20] Miyafuji H., Komai K., Kanbayashi T. (2017). Development of quantification method for lignin content in woody biomass by Raman micro-spectroscopy. Vib. Spectrosc..

[21] Lähdetie A. (2013). Wood Biomass Characterization by Raman Spectroscopy. Doctoral Dissertation.

[22] Toscano G., Maceratesi V., Leoni E., Stipa P., Laudadio E., Sabbatini S. (2022). FTIR spectroscopy for determination of the raw materials used in wood pellet production. Fuel.

[23] Jesus E., Franca T., Calvani C., Lacerda M., Gonçalves D., Oliveira S.L., Marangoni B., Cena C. (2024). Making wood inspection easier: FTIR spectroscopy and machine learning for Brazilian native commercial wood species identification. RSC Adv..

[24] Agarwal U.P., Reiner R.R., Ralph S.A. (2013). Estimation of Cellulose Crystallinity of Lignocelluloses Using Near-IR FT-Raman Spectroscopy and Comparison of the Raman and Segal-WAXS Methods. J. Agric. Food Chem..

[25] Duca D., Pizzi A., Rossini G., Mengarelli C., Foppa Pedretti E., Mancini M. (2016). Prediction of Hardwood and Softwood Contents in Blends of Wood Powders Using Mid-Infrared Spectroscopy. Energy Fuels.

[26] Gao W., Shu T., Liu Q., Ling S., Guan Y., Liu S., Zhou L. (2021). Predictive Modeling of Lignin Content for the Screening of Suitable Poplar Genotypes Based on Fourier Transform–Raman Spectrometry. ACS Omega.

[27] Remy N., Touboul D., Nicol E., Humbert S., Duma L., Lameiras P., Renault J.-H., Paës G. (2025). Chemical imaging of lignocellulosic biomass: Mapping plant chemistry. Biotechnol. Adv..

[28] Agarwal U.P. (2019). Analysis of cellulose and lignocellulose materials by Raman spectroscopy: A review of the current status. Molecules.

[29] Lupoi J.S., Gjersing E., Davis M.F. (2015). Evaluating lignocellulosic biomass, its derivatives, and downstream products with Raman spectroscopy. Front. Bioeng. Biotechnol..

[30] Leng W., He S., Lu B., Thirumalai R.V.K., Nayanathara R.O., Shi J., Zhang R., Zhang X. (2022). Raman imaging: An indispensable technique to comprehend the functionalization of lignocellulosic material. Int. J. Biol. Macromol..

[31] Sharma A., Garg S., Sharma V. (2024). ATR-FTIR Spectroscopy and Machine Learning for Sustainable Wood Sourcing and Species Identification: Applications to Wood Forensics. Microchem. J..

[32] Ralph J., Hatfield R.D., Sederoff R.R., MacKay J.J. (1998). Order and randomness in lignin and lignification: Is a new paradigm for lignification required?. Research Summaries USDFRC.

[33] Malavasi U.C., Davis A.S., Malavasi M.d.M. (2016). Lignin in woody plants under water stress: A review. Floresta Ambiente.

[34] Gerasimov V.A., Gurovich A.M., Kostrin D.K., Selivanov L.M., Simon V.A., Stuchenkov A.B., Paltcev A.V., Uhov A.A. (2016). Raman spectroscopy for identification of wood species. J. Phys. Conf. Ser..

[35] Sakrabani R., Mosca S., Liptak A., Burca G. (2025). Feasibility study on using combined tomography and spectroscopy techniques to evaluate the physical and chemical characteristics of organo-mineral fertilisers. Front. Sustain. Food Syst..

[36] Pinho R., Oliveira M., Teixeira B.M.M., da Silva Borges A.D. (2025). Evaluating quality and price dynamics of wood pellets in the Portuguese market: Impacts of geopolitical and economic factors. Energy Strategy Rev..

[37] Siyal A.A., Liu Y., Mao X., Ali B., Husaain S., Dai J., Zhang T., Fu J., Liu G. (2021). Characterization and quality analysis of wood pellets: Effect of pelletization and torrefaction process variables on quality of pellets. Biomass Convers. Biorefinery.

[38] Kamperidou V. (2022). Quality Analysis of Commercially Available Wood Pellets and Correlations between Pellets Characteristics. Energies.

[39] Demirbas A. (2004). Combustion characteristics of different biomass fuels. Prog. Energy Combust. Sci..

[40] da Silva S.B., Arantes M.D.C., de Andrade J.K.B., Andrade C.R., Carneiro A.d.C.O., Protásio T.d.P. (2020). Influence of physical and chemical compositions on the properties and energy use of lignocellulosic biomass pellets in Brazil. Renew. Energy.

[41] Picchio R., Latterini F., Venanzi R., Stefanoni W., Suardi A., Tocci D., Pari L. (2020). Pellet Production from Woody and Non-Woody Feedstocks: A Review on Biomass Quality Evaluation. Energies.

[42] Holub D., Pořízka P., Kizovský M., Prochazka D., Samek O., Kaiser J. (2022). The potential of combining laser-induced breakdown spectroscopy and Raman spectroscopy data for the analysis of wood samples. Spectrochim. Acta Part B At. Spectrosc..

[43] Börcsök Z., Pásztory Z. (2021). The role of lignin in wood working processes using elevated temperatures: An abbreviated literature survey. Eur. J. Wood Wood Prod..

[44] Demirbas A. (2002). Relationships between Heating Value and Lignin, Moisture, Ash and Extractive Contents of Biomass Fuels. Energy Explor. Exploit..

[45] Demirbaş A. (2001). Relationships between lignin contents and heating values of biomass. Energy Convers. Manag..

[46] Esteves B., Sen U., Pereira H. (2023). Influence of Chemical Composition on Heating Value of Biomass: A Review and Bibliometric Analysis. Energies.

[47] Agarwal U.P. (2006). Raman imaging to investigate ultrastructure and composition of plant cell walls: Distribution of lignin and cellulose in black spruce wood (*Picea mariana*). Planta.

[48] Vitek P., Klem K., Urban O. (2017). Application of Raman spectroscopy to analyse lignin/cellulose ratio in tree rings. Beskydy.

[49] Rebrošová K., Šiler M., Samek O., Růžička F., Bernatová S., Ježek J., Zemánek P., Holá V. (2017). Differentiation between *Staphylococcus aureus* and *Staphylococcus epidermidis* strains using Raman spectroscopy. Future Microbiol..

[50] Lasalvia M., Capozzi V., Perna G. (2022). A Comparison of PCA-LDA and PLS-DA Techniques for Classification of Vibrational Spectra. Appl. Sci..

[51] Thiffault E., Barrette J., Blanchet P., Nguyen Q.N., Adjalle K. (2019). Optimizing Quality of Wood Pellets Made of Hardwood Processing Residues. Forests.

[52] Raman Base: Open Online Database of Raman Spectra. Institute of Scientific Instruments of the Czech Academy of Sciences, Brno, Czech Republic. http://www.ramanbase.org.

